# Bee venom as an alternative for antibiotics against *Staphylococcus aureus* infections

**DOI:** 10.1038/s41598-023-33536-x

**Published:** 2023-04-20

**Authors:** Ahmed Sameh, Amr A. Gouda, Esraa Elmligy, Hossam Hatem, Salma S. Sadek, Osama Ahmed, Azza El Amir

**Affiliations:** 1 Zoology Department, Faculty of Science, Cairo University, Giza, 12613 Egypt; 2 Biotechnology Deptartment, Faculty of Science, Cairo University, Giza, 12613 Egypt

**Keywords:** Biological models, Drug development, Immunology, Diseases

## Abstract

The misuse of antibiotics has led to antibiotic-resistant bacterial strains, making it even harder to combat and eliminate their infections. *Staphylococcus aureus* causes various adverse infections and diseases, including skin abscesses, bloodstream infections, pneumonia, and joint infections. In this study, we aimed to test the cytotoxic and antibacterial effects of bee venom-loaded chitosan nanoparticles (BV-loaded CS-NPs) in comparison to gamma-irradiated BV and native BV from *Apis mellifera.* The physiochemical characterizations of our treatments were determined by Fourier Transform Infrared Spectroscopy (FTIR), Transmission Electron Microscope (TEM), zeta-potential, release rate, and Encapsulation Efficiency (EE). Our study was conducted on both levels, *in-vitro* and *in-vivo.* For the *in-vitro* study, a bacterial model of *Staphylococcus aureus* with an ATCC number of 6538 was grown in tryptic soy agar (TSA) medium, and the inhibition zones of our drug candidates were measured with the appropriate statistical analysis performed. For the *in-vivo* study, levels of aspartate aminotransferase (AST), alanine aminotransferase (ALT), Creatinine, Urea, and interleukin 6 (IL-6) were analyzed. BV-loaded CS-NPs showed relatively better results than the other alternatives, which are native BV and gamma-irradiated BV. The results showed that the antibacterial effect of BV-loaded CS-NPs was greater than the alternatives. Furthermore, its cytotoxic effect was far less than the native and irradiated bee venom. These outcomes ensure that loading BV on CS-NPs makes it a promising drug candidate for an antibiotic alternative with minimal cytotoxicity and enhanced antibacterial activity.

## Introduction

Antibiotic usage in massive quantities may generate a high selection pressure on the human and natural microbial systems^1^. The effect of antibiotics on the microbial systems may cause genetic or mutational changes in typically sensitive bacteria allowing the bacteria to live and grow as antibiotic-resistant bacteria (ARB)^2^, expressing antibiotic-resistant genes^3^. ARB is the main cause of the death of more than 1.27 million people every year. This number is expected to rise if no serious actions are taken^4^. *Staphylococcus aureus* is one of the most vicious strains that have developed antibiotic resistance*.* It is a bacterium well known for causing various harmful diseases, ranging from skin abscesses, joint infections, and pneumonia to bloodstream infections^5^. Antibiotic-resistant *S. aureus* can lead to more complicated conditions than the wild strain. Treatment of these antibiotic-resistant bacteria can lead to huge advancements against adverse infections^6^. Due to these reasons, searching for natural alternatives for antibiotics, such as natural products^6^, essential oils^7^, and venoms, especially bee venom (BV), is very crucial^8^.

BV produced by honeybees (*Apis mellifera*) is a bitter, odorless, colorless acidic liquid pH (4.5–5.5)^9^ and contains a mixture of different active molecules such as peptides including melittin, apamin, adolapin, mast cell degranulating peptide, and enzymes (phospholipase A2 (PLA2) and hyaluronidase)^10^, as well as other components, such as histamine, dopamine, and epinephrine^11^. Melittin is the major component of BV and accounts for 50% of dry venom. The second major component is PLA2, which accounts for 12% of dry venom^12^. As a result of these components, BV has antimicrobial and anti-inflammatory characteristics and is currently used against many diseases like rheumatoid, Alzheimer's, amyotrophic lateral sclerosis, and cancer^11^.

However, this significant therapeutic potential of BV is limited to a specific dosage (1–3 μg/ml) above which it shows cytotoxic features^13,14^. There are various methods used to reduce and eliminate the cytotoxic effect of BV. Our two chosen approaches were exposing BV to gamma radiation and loading the venom on nanoparticles (NPs). Ionizing radiation, specifically gamma radiation, has been widely tested on venoms in the past decade; it mainly depends on inducing structural changes in BV components. Previous studies showed that gamma radiation could have caused conformational changes, such as molecule chain unfolding, hence modifying the hydrophobic group exposure^15^. As well as eliminating some toxic effects of BV, such as hemolysis, without affecting its bioactivity, irradiated venom has lower hemolytic and PLA2 activity than natural venom^16^.

In addition to using gamma-irradiated BV, there is another way to reduce BV cytotoxicity using drug delivery systems, especially NPs. NPs have many advantages, including high drug capacity, sustainable drug release, high stability, the feasibility of including a hydrophilic or a hydrophobic drug, easy preparation, and low cost^17^. We decided to use chitosan nanoparticles (CS-NPs) for many reasons, including less requirement for harsh solvents, stability, and easy preparation. CS-NP has been used in various fields and applications^18^. It is a natural antimicrobial agent targeting gastrointestinal diseases^19^.

Therefore, this study aimed to compare the cytotoxic effect of CS-NPs, BV-loaded CS-NPs, and irradiated BV. In addition, we aimed to test the antibacterial effect of each of the candidates mentioned previously and their potential as an alternative for antibiotics against *S. aureus* infection.

## Materials and methods

### Materials

Low molecular weight (3600) white powder Chitosan (CS) was used and purchased from Alamia Company for Chemicals, Egypt (Cat No.: C0108), sodium tripolyphosphate (Na-TPP) anhydrous, white powder (Lot No.: F19H558) was used and purchased from (Advent, Egypt). Bradford reagent obtained from Takara Bio Inc., USA (Cat No.: T9310A). Bovine Serum Albumin (BSA) was purchased from BioWest, Nuaillé, France (Cat No.: P6154-10GR). White powder Tryptic Soy Broth (TSB) was purchased from Becto, Dickinson, and Company sparks, USA. (Lot No.: 2166207). Agar was purchased from Alpha Chemika, India. (Lot No.: ES00334). Ampicillin (Cat No.: CT0003B) and Erythromycin (Cat No.: CT0020B) were purchased from ThermoFisher, USA. Mouse IL-6 ELISA Kit (Cat No.: MBS824703), Blood Urea Nitrogen Microplate Assay Kit (Cat No.: MBS508399), and Mouse Creatinine (CR) ELISA Kit (Cat No.: MBS3805479) were purchased from MyBioSource San Diego (USA). Other reagents and chemicals used were of analytical grade.

### Bee venom (BV)

BV from *A. melifera* (10 g) was received in its lyophilized powder from the Ministry of Agriculture, Egypt, and stored. Half of the amount of the BV was irradiated with a ^60^Co gamma radiation source at a dosage rate of 0.7 KGy/hr at the National Center for Radiation and Technology (NCRRT, Egypt). Dry venom samples were irradiated at 20 KGy.

### Preparation of CS-NPs and BV- loaded CS-NPs

CS-NPs were prepared using the ionic gelation method based on electrostatic interaction between anions and cations^20^. Briefly, the chitosan powder of 400 mg was dissolved in 200 ml of 2% acetic acid (2 mg/ml) (pH 5.4). In parallel, 100 mg of Na-TPP was dissolved in 100 ml of distilled water (1 mg/ml). Afterward, the Na-TPP solution was titrated drop-wisely against the previously prepared CS solution at room temperature, using a magnetic stirrer (1000 rpm, 60 min). Lastly, the CS-NPs were precipitated using a cooling centrifuge (12,000 rpm, 8 °C, 30 min), and the pellets containing NPs were collected into a sterile falcon, freeze-dried, and stored at 20 °C. For BV-loaded CS-NPs, BV (550 µg/ml) was added to the Na-TPP solution and titrated against the CS solution.

### Characterization

Physiochemical characterization tests were done on free BV, gamma-irradiated BV, unloaded CS-NPs, and BV-loaded CS-NPs.

#### Fourier transform infrared spectroscopy (FTIR)

FTIR was performed to confirm the identity of components, the difference in functional groups, and the changes between free BV, irradiated BV, CS-NPs, and BV-loaded CS-NPs. The device used was JASCO 1, with a resolution Delta X of 0.964233 cm^−1^.

#### Transmission electron microscope (TEM)

TEM was done to determine the morphology and particle size of both CS-NPs and BV-loaded CS-NPs by using JEOL- JEM.1010.

#### Zeta-potential and particle size distribution

The prepared NPs were analyzed for their particle size and size distribution in terms of the average volume diameters and polydispersity index (PDI) by photon correlation spectroscopy using the particle size analyzer Dynamic Light Scattering (DLS) (Zetasizer Nano ZN, Malvern Panalytical Ltd, UK) at a fixed angle of 173°. Samples were analyzed in triplicates the same equipment measured zeta potential to detect the repulsion between NPs^21^.

#### Determination of encapsulation efficiency (EE)

EE is the method that measures the amount of BV uptaken by the CS-NPs, and it was carried out using the Bradford protein assay. The standard curve was drawn using BSA. The solutions of both BV-loaded CS-NPs and unloaded CS-NPs were centrifuged. The supernatant was collected, different BSA concentrations were prepared, and the Bradford reagent was added accordingly. Hence, using a 96-well microtiter plate, the amount of BV loaded on the NPs was determined^22^. The EE was calculated according to the equation below^1^:

1 $$ {\text{EE }}\left( {\text{\% }} \right) = { }\frac{{{\text{Total}}\;{\text{amount }}\;{\text{of}}\;{\text{ BV}}\;{\text{ initially}}\;{\text{added}} - {\text{amount }}\;{\text{of}}\;{\text{BV}}\;{\text{detected}}\;{\text{in}}\;{\text{supernatant}}}}{{{\text{Total }}\;{\text{amount }}\;{\text{of}}\;{\text{ BV}}\;{\text{ initially}}\;{\text{ added}}}} \times 100 $$

#### *In-vitro* release rate of BV-loaded CS-NPs

0.1 g of lyophilized BV-loaded CS-NPs was dissolved in 3 ml of phosphate buffer saline (PBS) (0.2 mol/L) at a pH of 7.4. After the preparation of the solution, it was divided into different falcons, each with varying intervals of time (1, 2, 4, 8, 16, 24, 30, and 42 h), and incubated in a water bath (37 °C). Then, the samples were centrifuged at 20,000 × g/9900 rpm (14 °C for 30 min) at determined time intervals. Lastly, the amount of BV released was assessed with Bradford Assay, and BioTek Powerwave XS2 Plate Reader detected the color change^22^.

### Bacterial model

The bacteria used was *S. aureus* with ATCC 6538. It was cultured in TSB for 18 h at 37 °C until the suspension reached OD of 0.5, and the number of bacteria was adjusted to ~ 1.5 × 10^9^ CFU/mL at 600 nm^23^.

### *In-vitro* study

The bacteria was inoculated in Tryptic Soya Agar (TSA) media, and its antibacterial effect was tested by the disc diffusion method. The treatments (BV, irradiated BV, CS-NPs, BV-loaded CS-NPs) were used in a concentration of (1 g/L in PBS) the concentrations were adjusted according to the LD_50_ of BV that was determined by our previous study^23^ and were dissolved in 5 ml PBS, lightly vortexed, discs were soaked in it, then added uniformly to the plate. Ampicillin (10 µg/disc) and Erythromycin (15 µg/disc) discs were used as controls. The disc diffusion study was repeated 3 times to confirm reproducible data.

### *In-vivo* study

#### Ethical consideration

All the experimental procedures were performed under the international guidelines for the care and use of laboratory animals. The experimental animal protocol was approved (Protocol Number CUIF5521) by the Institutional Animal Care and Use Committee of Cairo University (CUIACUC) (Giza, Egypt). Our study experiments were performed in accordance with ARRIVE guidelines.

#### Treatments dosage preparation

Mice infection was carried out Intraperitoneally *(i.p.)* with freshly prepared bacterial suspension ~ 1.5 × 10^8^ CFU/mL, and the treatments started 3 h post-infection. All treatments were injected *i.p.* every 24-h (triple dose). The injected BV dose was 9.02 µg/g mouse weight, according to a previous study^23^. Suspension of BV-loaded CS-NPs was dissolved in PBS (1 mg/ml) and vortexed to prepare an equivalent volume to the BV dosage according to the NPs' Encapsulation Efficiency. Accordingly, similar volumes of BV-loaded CS-NPs were used to inject CS-NPs suspension (1 mg/ml). The antibiotic was also injected at the same dose.

#### Experimental design

Seventy-seven healthy Swiss male albino mice, aged 8–10 weeks (25–35 g), were obtained from the animal house of The Nile Company for Pharmaceuticals & Chemical Industries, Egypt, and given proper housing in terms of diet, water, day/night cycles, and healthy environmental conditions.

The animals were divided into 11 groups (7 mice/group), Gp.1: Mice injected with PBS as a control group. Gp.2: Mice injected with BV. Gp.3: Mice injected with gamma-irradiated BV. Gp.4: Mice injected with unloaded CS-NPs. Gp.5: Mice injected with BV- loaded CS-NPs. Gp.6: Mice infected with bacteria. Gp.7: Infected mice treated with BV. Gp.8: Infected mice treated with gamma-irradiated BV. Gp.9: Infected mice treated with unloaded CS-NPs. Gp.10: Infected mice treated with BV–loaded CS-NPs. Gp.11: Infected mice treated with a commercial antibiotic (sulbacef).

#### Sample collection and biochemical and haematological analysis

All mice were euthanized one-week post-infection with sodium pentobarbital. Blood samples were collected in two different types of tubes, ethylenediaminetetraacetic acid (EDTA) tubes for haematological investigations and serum tubes for biochemical analysis of liver function represented in alanine transaminase (ALT) and aspartate transaminase (AST), Renal function urea and creatinine were measured using (Cat No.: MBS508399), (Cat No.: MBS3805479), and interleukin 6 (IL-6) using (Cat No.: MBS824703), purchased from MyBioSource San Diego (USA), The Mouse IL-6 sandwich ELISA was done according to the protocol of the kit.

#### Histopathological analysis

After euthanasia, the liver, kidney, thymus, and spleen samples were collected from all experimental groups, fixed in 10% buffered formalin, embedded in paraffin, sectioned into 4–5 µm sections, deparaffinized in xylene, hydrated, and stained with hematoxylin and eosin (H&E), and examined under a light microscope.

### Data analysis

The IBM statistical package for the Social Sciences, version 25, was used to analyze the data (copyright by IBM SPSS software, US). For NPs characterization experiments, one sample T-test was applied. For in vitro and in vivo experiments, a one-way Analysis of variance (ANOVA) was used. The smallest level of significance was *P* < *0.05*. Post hoc ANOVA (Tukey's homogeneity test) was used to test the differences and similarities between the experimental groups. All results were represented as a mean ± standard error of the mean (SEM). GraphPad Prism version 8 was used to plot the data (Graph Pad Software Inc., San Diego, CA).

## Results

### FTIR analysis

**Figure 1:**
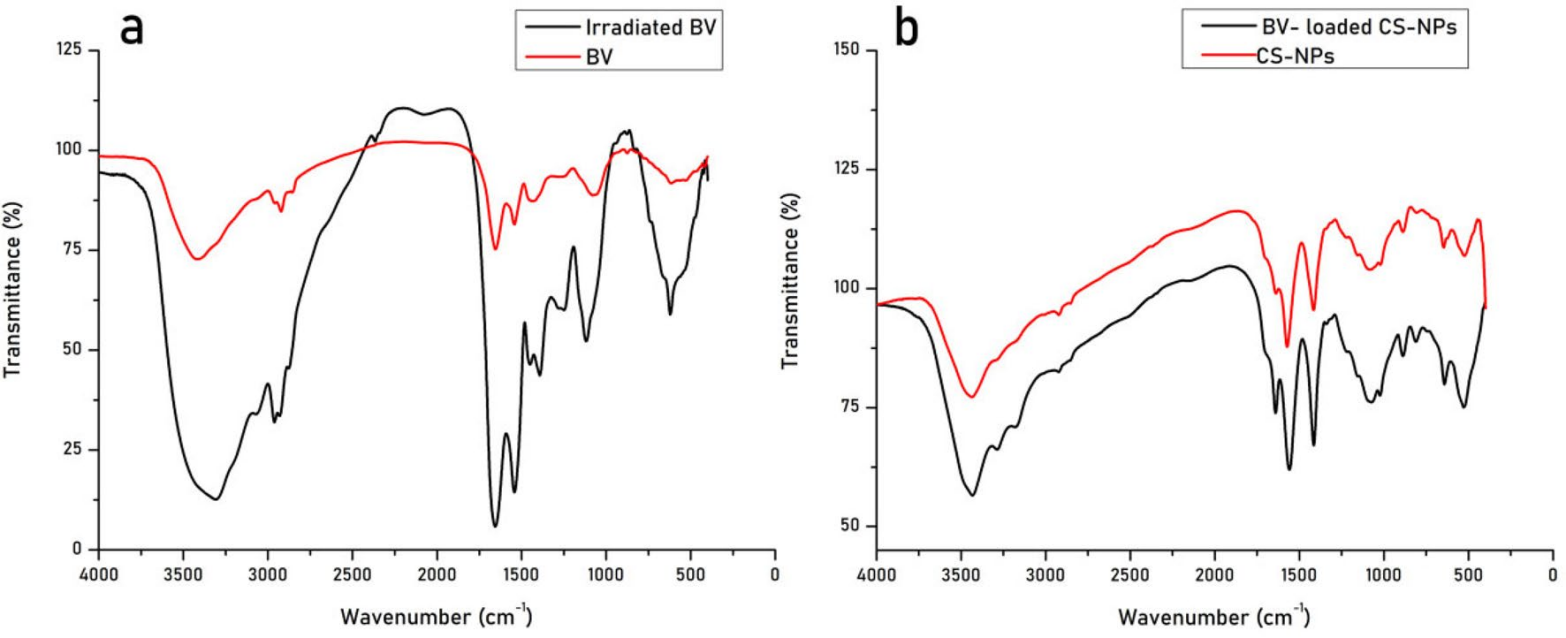
FTIR spectra of (**a**) 20 kGy gamma-irradiated BV compared to BV and (**b**) CS-NPs compared to BV-loaded CS-NPs. -BV: Bee venom, CS-NPs: Chitosan nanoparticles, BV-loaded CS-NPs: Bee venom-loaded Chitosan nanoparticles.

The main purpose was to identify the specific peaks for peptides in the BV secondary structure and observe any changes in those peaks in FTIR spectra of irradiated BV. The FTIR spectra of both are shown in (Fig. 1a). In addition to comparing CS-NPs and BV-loaded CS-NPs as shown in Fig. 1b. According to a chemical profiling study, BV showed normal peaks that provide the basic characteristic FTIR spectra of natural venom. The analysis shows significant peaks at approximately 3400 cm^−1^ and 2940 cm^−1^, while the fingerprint region for BV lies between 1700 and 600 cm^−1^. This region showed bands with sharp peaks at approximately 1680 cm^-1^ and 1500 cm^−1^, a peak at nearly 1400 cm^−1^, and 3 bands at approximately 1050, 945, and 630 cm^−1^. All the peaks observed in the spectra of BV are found in the irradiated BV, but they show alteration and have quite lower intensity.

The FTIR spectrum of CS-NPs was characterized by three bands (Fig. 1b). A band at 3500–3300 cm^−1^ related to NH_2_ and O–H stretching vibration, a band at 1633 cm^-1^ refers to NH_2_ bending vibration, and a band at 1150 cm^−1^ corresponds to C–O–C asymmetric stretching vibration. The FTIR spectra of BV-loaded CS-NPs exhibit a distinctive sharp band at 1642 cm^-1^, and a peak at 560 cm^−1^ was observed in both loaded and unloaded NPs.

### TEM analysis of NPs

**Figure 2:**
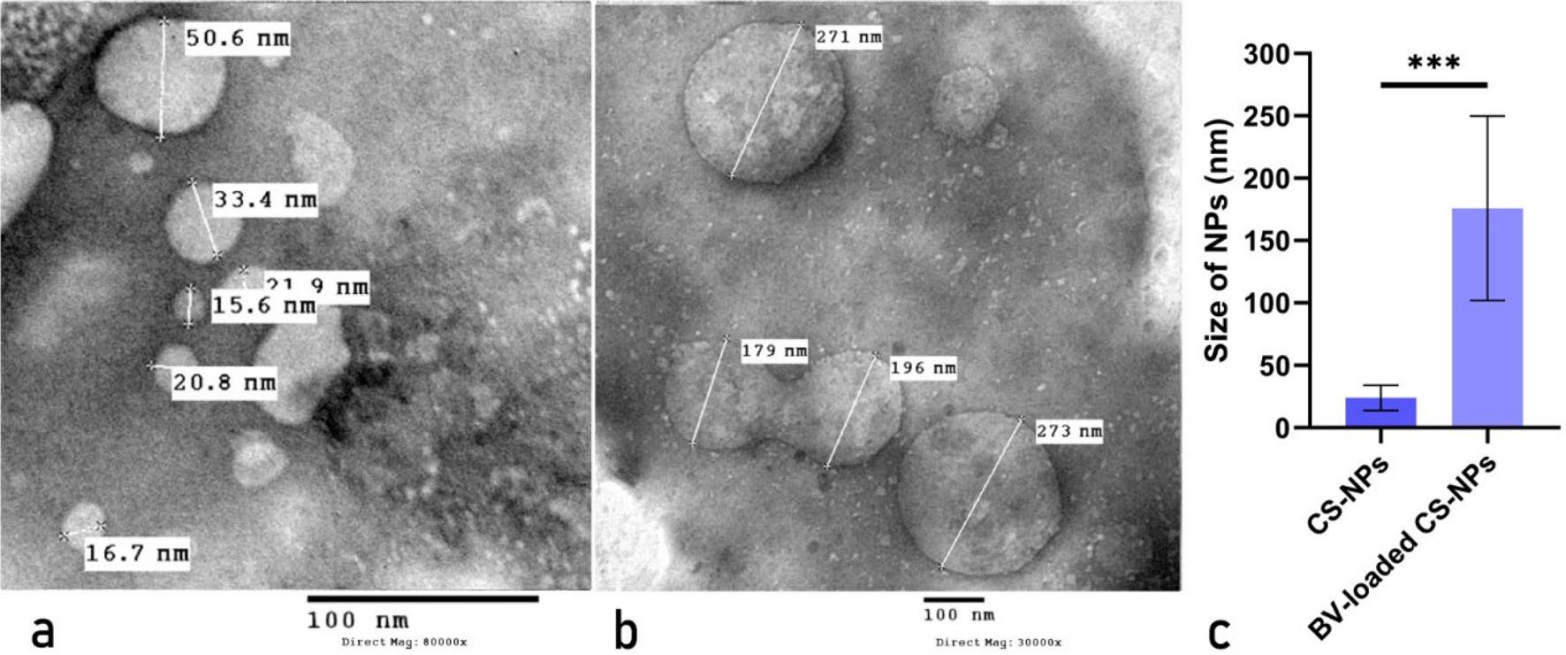
Particle size distribution of (**a**) CS-NPs and (**b**) BV-loaded CS-NPs obtained by (TEM). (**c**) The sizes of prepared NPs. Data were analyzed using a one-sample t-test and represented as Mean ± Standard Error of mean (SEM). (***) is considered highly significant *P* < 0*.*0001. -CS-NPs: Chitosan nanoparticles, BV-loaded CS-NPs: Bee venom-loaded Chitosan nanoparticles.

TEM analysis was implemented to study the particle size distribution and morphology of CS-NPs and BV-loaded CS-NPs. The result showed that CS-NPs possess an average size of 24.03 ± 2.22 nm with a smooth surface, while the BV-loaded CS-NPs had an average size of 175.85 ± 23.41 nm, as shown in (Fig. 2).

### Zeta potential and PDI

**Figure 3:**
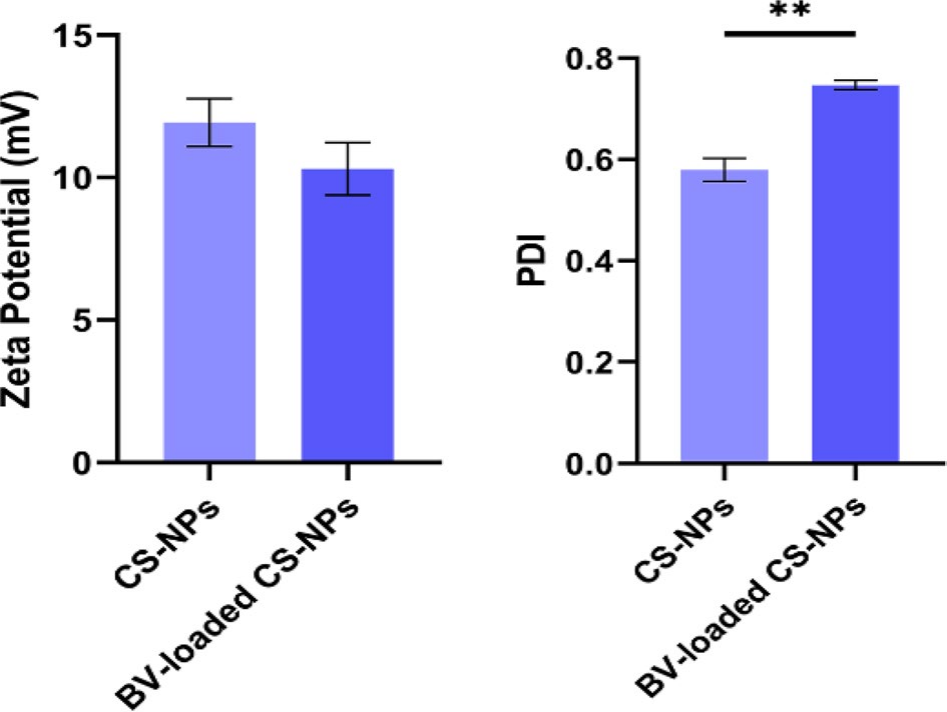
Surface net charge and NPs' uniformity in the solution of CS-NPs and BV-loaded CS-NPs were measured using zeta potential and PDI. Data were analyzed using a one-sample T-test and represented as Mean ± Standard Error of mean (SEM). (**) is considered significant *P* < 0.01. -CSNPs: Chitosan nanoparticles, BV-loaded CS-NPs: Bee venom-loaded Chitosan nanoparticles.

The determined zeta potential and PDI of both CS-NPs and BV-loaded CS-NPs are described in (Fig. 3). A decrease in the zeta potential was observed in BV-loaded CS-NPs, and a significant increase (*P* < 0.001) in the PDI value was observed in BV-loaded CS-NPs compared to the CS-NPs.

### Encapsulation efficiency (EE) of BV-loaded CS-NPs

The optimum concentration of BV and CS for the preparation of BV-loaded CS-NPs were 550 µg/mL and 2 mg/ml, respectively. The results ensured that by yielding loaded nanoparticles with a high EE of 96.44%.

### *In-vitro* release rate of BV-loaded CS-NPs

**Figure 4:**
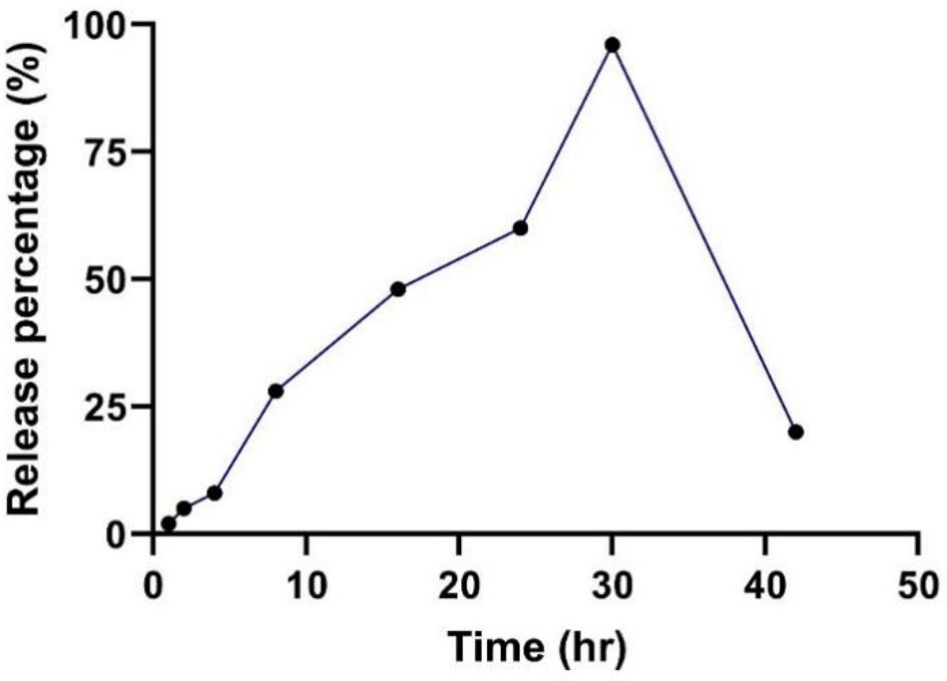
Releasing profile of BV from BV-loaded CS-NPs at different time intervals. BV: Bee venom, BV-loaded CS-NPs: Bee venom-loaded Chitosan nanoparticles.

As shown in (Fig. 4), the amount of released BV from CS-NPs increased exponentially over time until it reached its optimum release at 30 h. After that specific interval, the amount of BV released began to decrease.

### In vitro study

**Figure 5:**
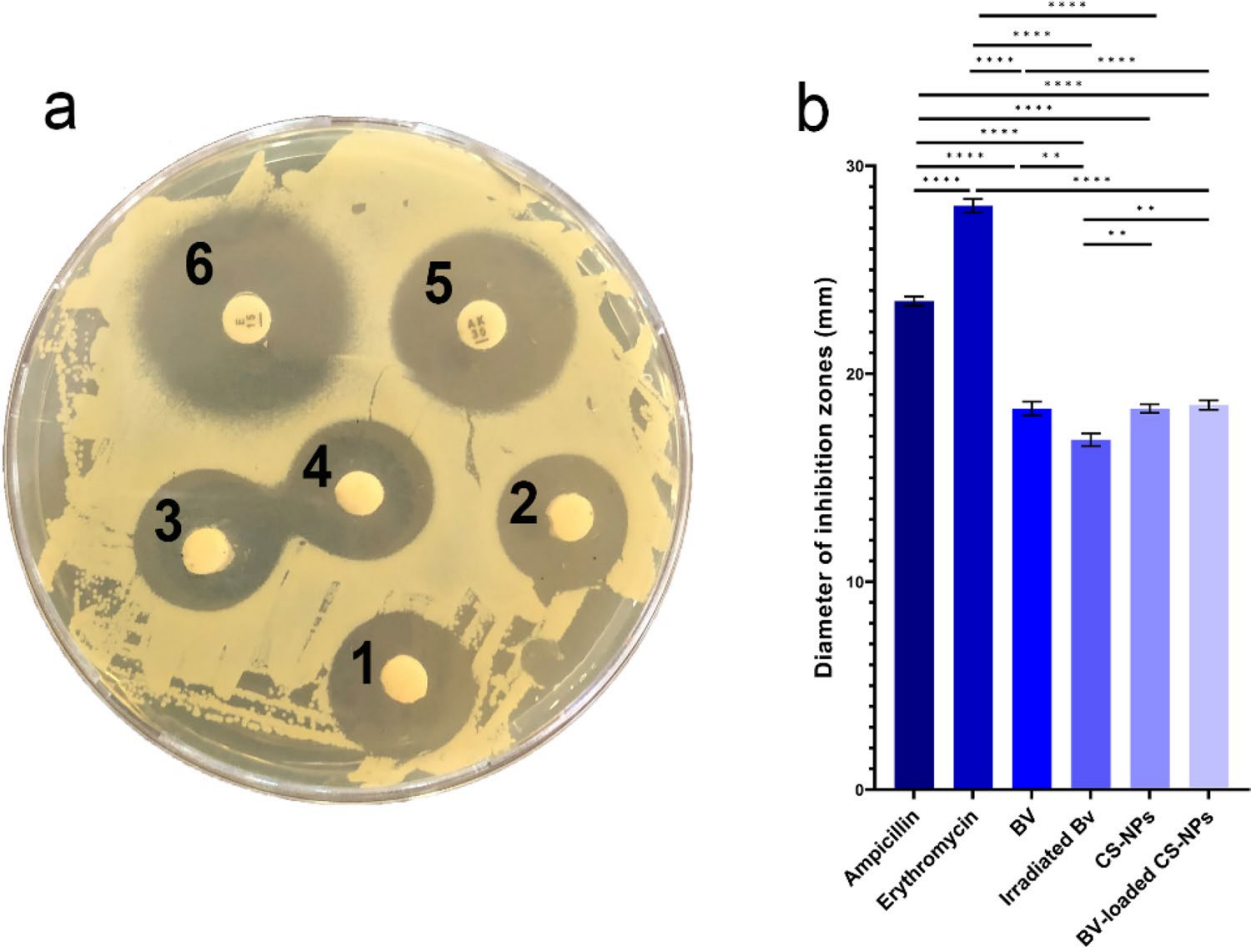
(**a**) TSA plate showing antibacterial activity of (1) BV, (2) Irradiated BV, (3) CS-NPs, (4) BV-loaded CS-NPs, (5) ampicillin (10 µg/disk), and (6) Erythromycin (15 µg/disk) against *S. aureus*. (**b**) Comparing the diameter of inhibition zones of BV, irradiated BV, CS-NPs, and BV-loaded CS-NPs against Ampicillin and Erythromycin. Data were analyzed using one-way ANOVA and represented as Mean ± Standard Error of mean (SEM). (**) is considered highly significant *P* < 0.01, (****) is considered highly significant *P* < 0.0001. -TSA: Tryptic Soy Agar, BV: Bee venom, CS-NPs: Chitosan nanoparticles, BV-loaded CS-NPs: Bee venom loaded Chitosan nanoparticles.

The inhibition zone diameter was measured using the disc diffusion method to compare the antibacterial effect of the treatments against *S. aureus*, as shown in (Fig. 5a). The treatments gave different inhibition zones, as described in (Fig. 5b).

### Hematological investigations

**Figure 6:**
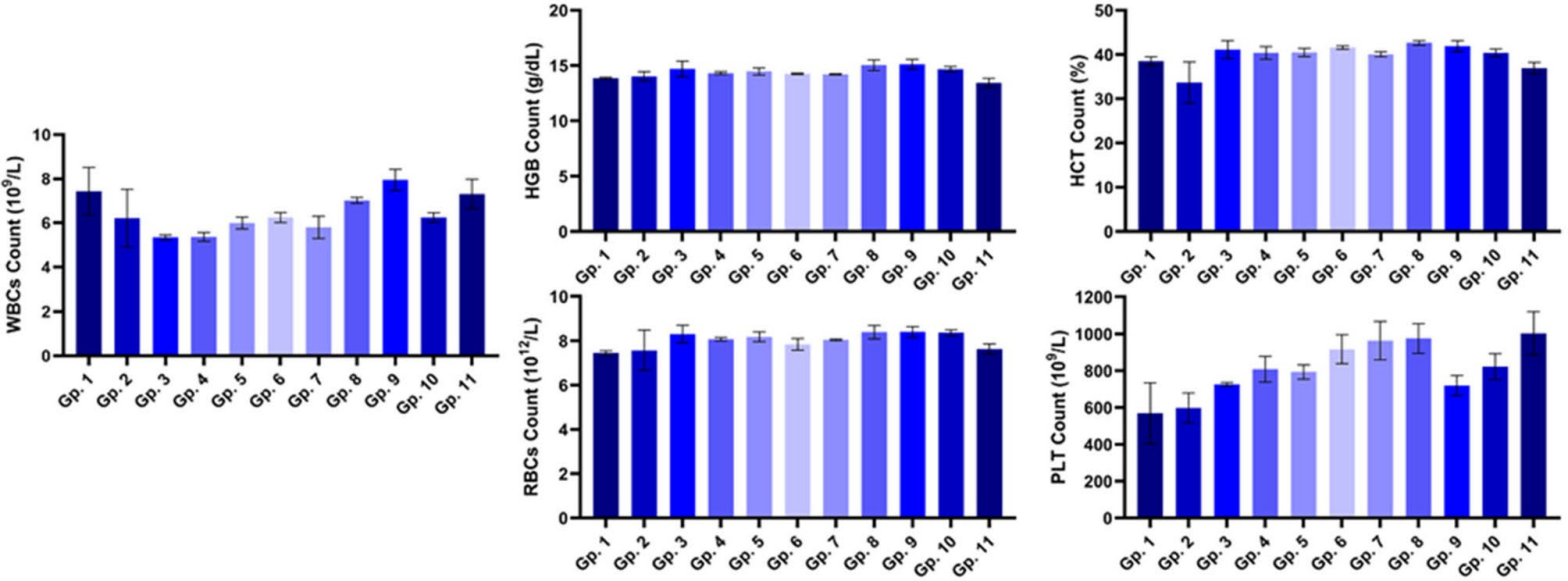
Effect of BV, irradiated BV, CS-NPs, and BV-loaded CS-NPs on WBCs count, HGB count, RBCs count, HCT count, and PLT count in studied groups. Gp.1: Mice injected with PBS as a control group, Gp.2: Mice injected with BV, Gp.3: Mice injected with irradiated BV, Gp.4: Mice injected with CS-NPs, Gp.5: Mice injected with BV-loaded CS-NPs, Gp.6: Infected mice with *S. aureus*, Gp.7: Infected mice treated with BV, Gp.8: Infected mice treated with irradiated BV, Gp.9: Infected mice treated with CS-NPs, Gp.10: Infected mice treated with BV–loaded CS-NPs, Gp.11: Infected mice treated with an antibiotic. Data were analyzed using one-way ANOVA and represented as Mean ± Standard Error of mean (SEM). -BV: Bee venom, CS-NPs: Chitosan nanoparticles, BV-loaded CS-NPs: Bee venom loaded Chitosan nanoparticles, WBCs: White blood cells, HGB: Hemoglobin, RBCs: Red blood cells, PLT: Platelets, HCT: Hematocrit.

Hematological analysis of blood samples showed that all treatments free and irradiated BV, CS-NPs, and BV-loaded CS-NPs exhibited a decrease in both WBCs and HCT count while showing a slight rise in PLT count. On the other hand, the infection group (Gp.6) had a significant drop in WBC count and PLT count. At the same time, treatment of bacterial infection with free and irradiated BV (Gp.7 & 8) had the lowest WBC count and the highest PLT level. It was obvious from the results (Fig. 6) that CS-NPs and BV-loaded CS-NPs (Gp. 9 &10) have a noticeable role in the treatment of bacterial infection by adjusting the count of WBCs and PLT when compared to the control group (Gp. 1). Treatment with antibiotic (Gp.11) causes an increase in PLT count.

### Biochemical investigations

In this study, the mice group injected with irradiated BV (Gp.3) or BV-loaded CS-NPs (Gp.5) showed an increase in ALT, AST, and creatinine levels and a decrease in urea levels compared to the control group (Gp.1). On the other side, the infected groups with *S. aureus*. Furthermore, infected untreated showed a significant increase (*P* < 0.01) in ALT levels and a marked increment but not significant in AST, creatinine, and urea levels.

**Figure 7:**
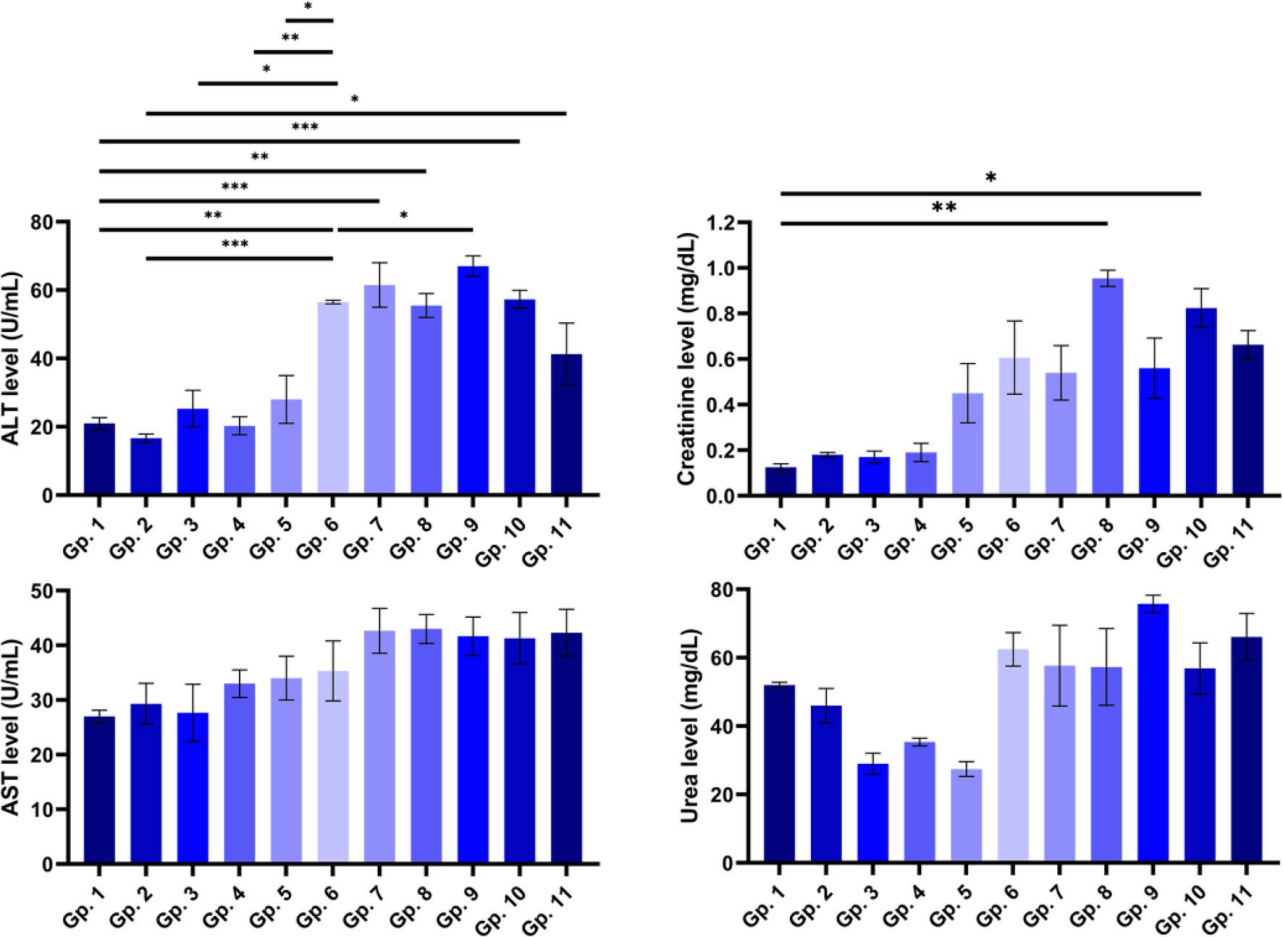
Effect of BV, irradiated BV, CS-NPs, and BV-loaded CS-NPs on ALT level, AST level, creatinine level, and urea level in studied groups. Gp.1: Mice injected with PBS as a control group, Gp.2: Mice injected with BV, Gp.3: Mice injected with irradiated BV, Gp.4: Mice injected with CS-NPs, Gp.5: Mice injected with BV-loaded CS-NPs, Gp.6: Infected mice with *S. aureus*, Gp.7: Infected mice treated with BV, Gp.8: Infected mice treated with irradiated BV, Gp.9: Infected mice treated with CS-NPs, Gp.10: Infected mice treated with BV–loaded CS-NPs, Gp.11: Infected mice treated with the antibiotic. Data were analyzed using one-way ANOVA and represented as Mean ± Standard Error of mean (SEM). (*) is considered significant *P* < 0.05, (**) is considered highly significant *P* < 0.01, (***) is considered highly significant *P* < 0.001.

All the treated groups (BV-treated (Gp.7), irradiated BV-treated (Gp.8), CS-NPs treated group (Gp.9), BV-loaded CS-NPs treated (Gp.10), and treated group with antibiotic (Gp.11)) showed an increase in all parameters' levels compared to control. Interestingly, the enhancement of BV-loaded CS-NPs on the different parameters was similar to the treated group with the antibiotic (Fig. 7).

### Measurement of Interleukin 6 (IL-6)

**Figure 8:**
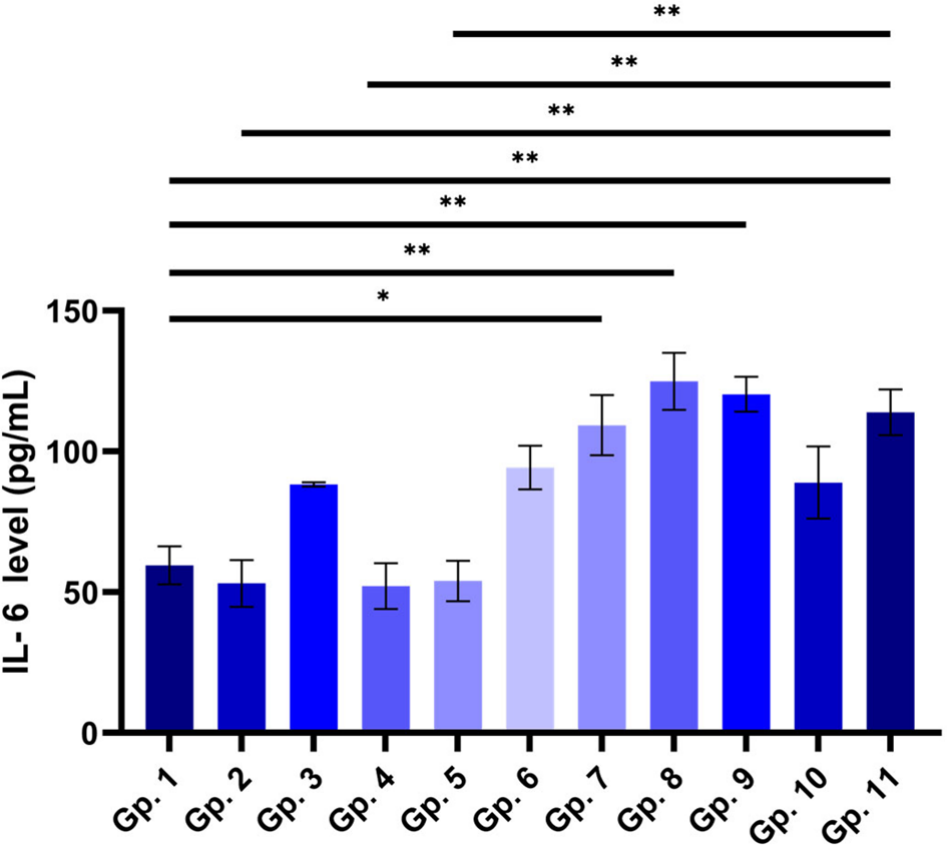
Effect of BV, irradiated BV, CS-NPs, and BV-loaded CS-NPs on IL-6 level in studied groups. Gp.1: Mice injected with PBS as a control group, Gp.2: Mice injected with BV, Gp.3: Mice injected with irradiated BV, Gp.4: Mice injected with CS-NPs, Gp.5: Mice injected with BV- loaded CS-NPs, Gp.6: Infected mice with S. aureus, Gp.7: Infected mice treated with BV, Gp.8: Infected mice treated with irradiated BV, Gp.9: Infected mice treated with CS-NPs, Gp.10: Infected mice treated with BV–loaded CS-NPs, Gp.11: Infected mice treated with an antibiotic. Data were analyzed using one-way ANOVA and represented as Mean ± Standard Error of mean (SEM). (*) is considered significant *P* < 0.05, (**) is considered highly significant *P* < 0.01.

A major rise in IL-6 levels was spotted, showing that the infection took place. Infected groups treated with BV (Gp.7), irradiated BV (Gp.8), or CS-NPs (Gp.9) showed elevated levels of IL-6 compared to the control group (Gp.1); they had much higher levels of IL-6. The BV-loaded CS-NPs treated group (Gp.10) was the only group to show a decrease in IL-6 levels when compared to the untreated infected group (Gp. 6) or antibiotic-treated group (Gp. 11) (Fig. 8).

### Histopathological analysis

#### Histopathological analysis of liver samples

Sections of the control liver had normal hepatocytes and veins. BV and irradiated BV groups showed mild congestion of the portal vein along with scattered apoptotic hepatocytes in the peri-portal area, in addition to the presence of mildly vacuolated cytoplasm in the BV group only. Meanwhile, both CS-NPs and BV-loaded CS-NPs groups had mildly dilated central veins and average hepatocytes. Still, the BV-loaded CS-NPs group had mild peri-venular inflammatory infiltrate.

**Figure 9:**
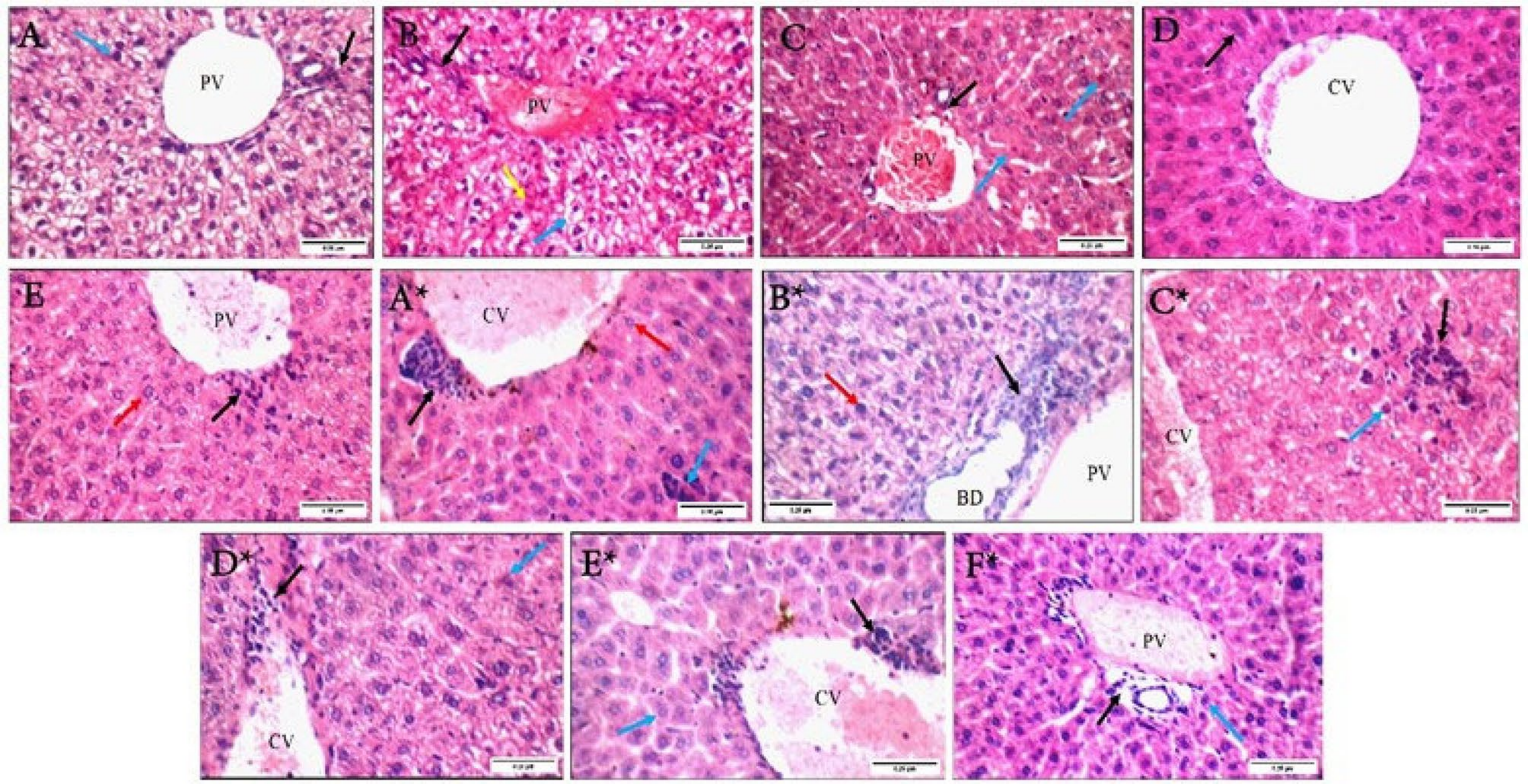
Liver sections of non-infected groups: (**A**) control group showing average portal tract (black arrow) with an average portal vein (PV), and average hepatocytes in peri-portal area (blue arrow), (**B**) BV group showing average portal tracts (black arrow) with a mildly congested portal vein (PV), and hepatocytes in peri-portal area showing mildly vacuolated cytoplasm (blue arrow) and scattered apoptosis (yellow arrow), (**C**) irradiated BV group showing average portal tracts (black arrow) with the mildly congested portal vein (PV), and scattered apoptotic hepatocytes in peri-portal area (red arrow), (**D**) CS-NPs group showing mildly dilated central vein (CV), and average hepatocytes in peri-venular area (black arrow), (**E**) BV-loaded CS-NPs showing mildly dilated central vein (CV) with mild peri-venular inflammatory infiltrate (black arrow), and average hepatocytes in peri- venular area (red arrow). Liver sections of infected groups with *S. aureus:* (A*) no treatment group showing mildly dilated congested central vein (CV) with mild peri-venular (black arrow) and intra-lobular inflammatory infiltrate (blue arrow), and average hepatocytes in peri-venular area (red arrow), (B*) BV group showing portal tracts with mild portal inflammatory infiltrate (black arrow), mildly congested portal vein (PV), and average hepatocytes in peri-portal area (red arrow), (C*) irradiated BV group showing mildly congested central vein (CV), and mild intra-lobular inflammatory infiltrate (black arrow) and scattered apoptotic hepatocytes (blue arrow), (D*) CS-NPs group showing mildly dilated central vein (CV) with mild peri-venular inflammatory infiltrate (black arrow), and scattered apoptotic hepatocytes in peri- venular area (blue arrow), (E*) BV-loaded CS-NPs showing markedly dilated congested central vein (CV) with mild peri-venular inflammatory infiltrate (black arrow), and average hepatocytes in peri-venular area (blue arrow), (F*) antibiotic group showing average portal tracts (black arrow) with average portal vein (PV), and average hepatocytes in peri-portal area (blue arrow) (H&E X 400). – BV: Bee venom, CS-NPs: Chitosan nanoparticles, BV-loaded CS-NPs: Bee venom loaded Chitosan nanoparticles, *S. aureus: Staphylococcus aureus*, H&E: Hematoxylin and Eosin.

On the other hand, all of the infected groups except the antibiotic-treated group seemed to have mild congestions and either peri-venular or intra-lobular mild inflammatory infiltrate. Both untreated and CS-NPs treated groups showed mildly dilated central veins, while the BV-loaded CS-NPs treatment showed markedly dilated central vein. Scattered apoptotic hepatocytes only appeared in the irradiated BV-treated sections (Fig. 9).

#### Histopathological analysis of kidney samples

**Figure 10:**
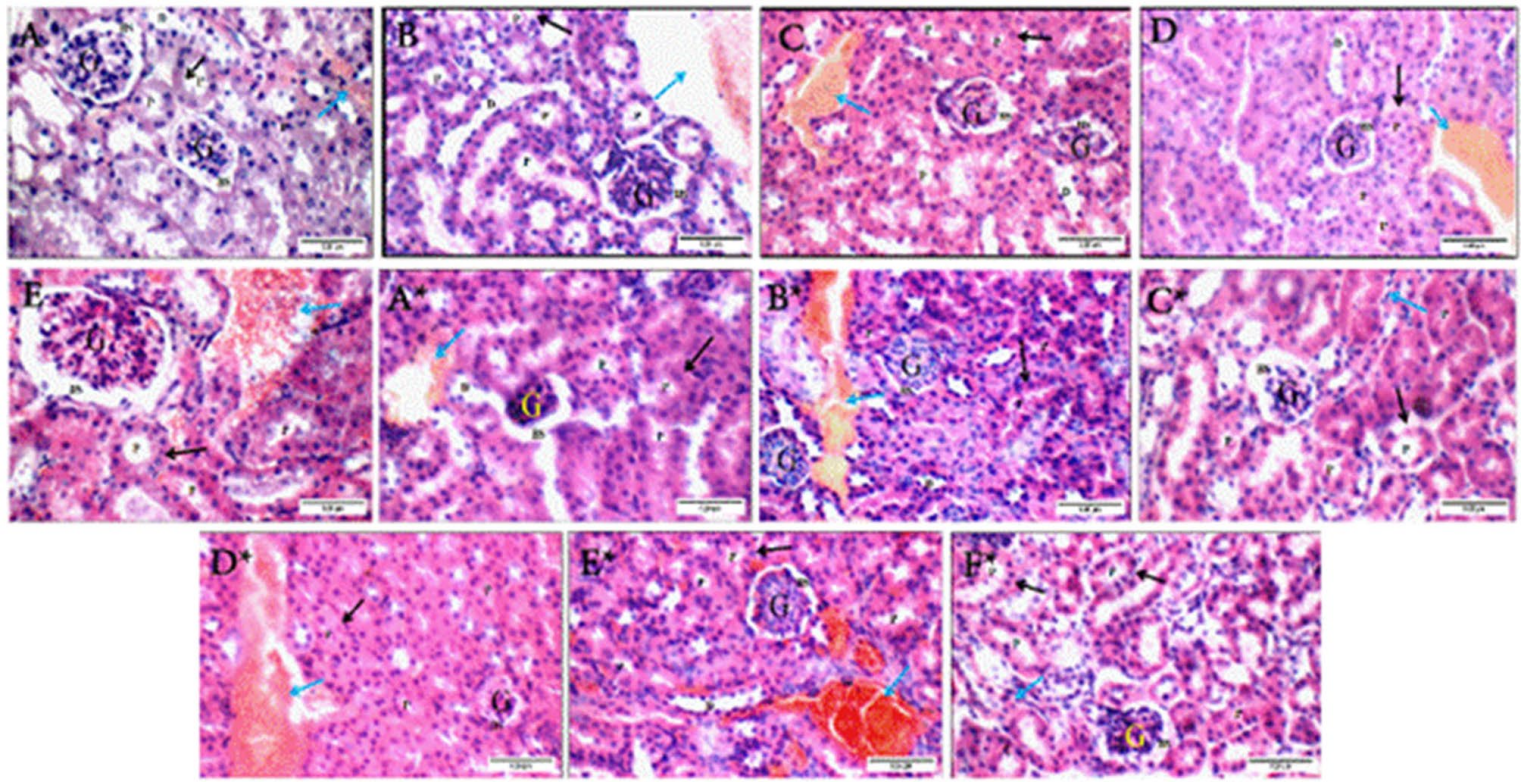
Kidney sections of non-infected groups: (**A**) control group showing average glomeruli (G) with average Bowman's spaces (BS), average proximal tubules (P) with preserved brush borders (black arrow), average distal tubules (D), and average interstitial blood vessels (blue arrow), (**B**) BV group showing average glomeruli (G) with average Bowman's spaces (BS), proximal tubules (P) with mildly edematous epithelial lining (black arrows), and markedly congested interstitial blood vessels (blue arrow), (**C**) irradiated BV group showing average glomeruli (G) with average Bowman's spaces (BS), proximal tubules (P) with average epithelial lining (black arrow), average distal tubules (D), and mildly congested interstitial blood vessels (blue arrow), (**D**) CS-NPs group showing average glomeruli (G) with average Bowman's spaces (BS), proximal tubules (P) with average epithelial lining (black arrows), and mildly congested interstitial blood vessels (blue arrow), (**E**) BV-loaded CS-NPs group showing average glomeruli (G) with average Bowman's spaces (BS), proximal tubules (P) with scattered apoptotic epithelial lining (black arrow), and markedly dilated congested blood vessels (blue arrow). Kidney sections of infected groups with *S. aureus*: (A*) no treatment group showing scattered atrophied glomeruli (G) with widened Bowman's spaces (BS), proximal tubules (P) with average epithelial lining (black arrow), and mildly congested blood vessels (blue arrow), (B*) BV group showing average glomeruli (G) with average Bowman's spaces (BS), proximal tubules (P) with average epithelial lining (black arrow), and mildly congested blood vessels (blue arrow), (C*) irradiated BV group showing atrophied glomeruli (G) with widened Bowman's spaces (BS), proximal tubules (P) with scattered apoptotic epithelial lining (black arrow), and average interstitium (blue arrow), (D*) CS-NPs group showing scattered small-sized glomeruli (G) with narrow Bowman's spaces (BS), proximal tubules (P) with scattered apoptotic epithelial lining (black arrow), and mildly congested blood vessels (blue arrow), (E*) BV-loaded CS-NPs group showing average glomeruli (G) with average Bowman's spaces (BS), proximal tubules (P) with average epithelial lining (black arrow), and mildly congested interstitial blood vessels (blue arrow), (F*) antibiotic group showing average glomeruli (G) with average Bowman's spaces (BS), proximal tubules (P) with average epithelial lining (black arrow), and average interstitium (blue arrow) (H&E X 400). – BV: Bee venom, CS-NPs: Chitosan nanoparticles, BV-loaded CS-NPs: Bee venom loaded Chitosan nanoparticles, *S. aureus: Staphylococcus aureus,* H&E: Hematoxylin and Eosin.

All kidney sections of non-infected samples showed average glomeruli with average Bowman's spaces as compared to control group (Fig. 10A), with minor changes in the BV group with mildly edematous epithelial lining and markedly congested interstitial blood vessels (Fig. 10B). Both irradiated BV and CS-NPs groups showed mildly congested interstitial blood vessels (Fig. 10C, D). The changes increased in the BV-loaded CS-NPs group with scattered apoptotic epithelial lining and markedly dilated congested blood vessels (Fig. 10E).

The infected groups with *S. aureus* showed different levels of kidney damage due to bacterial infection. The damage in the CS-NPs group was the highest, showing scattered small-sized glomeruli with narrow Bowman's spaces, scattered apoptotic epithelial lining, and mildly congested blood vessels (Fig. 10D*). The damage decreased in the irradiated BV group showing widened Bowman's spaces and scattered apoptotic epithelial lining (Fig. 10C*). The BV and BV-loaded CS-NPs groups showed average conditions similar to control and antibiotic groups with a minor change in the interstitial blood vessels, which were mildly congested (Fig. 10B*, E*, F*). These groups were compared to the infected group with *S. aureus* (Fig. 10A*).

#### Histopathological analysis of spleen samples

All non-infected groups showed average lymphoid follicles, but there was mild congestion in blood vessels of the BV group along with scattered apoptosis and similar congestion in the blood sinusoids of the CS-NPs group. Excess giant cells were also observed in the irradiated BV and CS-NPs groups. These observations leave the BV-loaded CS-NPs group the most similar to the control with both average blood sinusoids and scattered giant cells.

**Figure 11:**
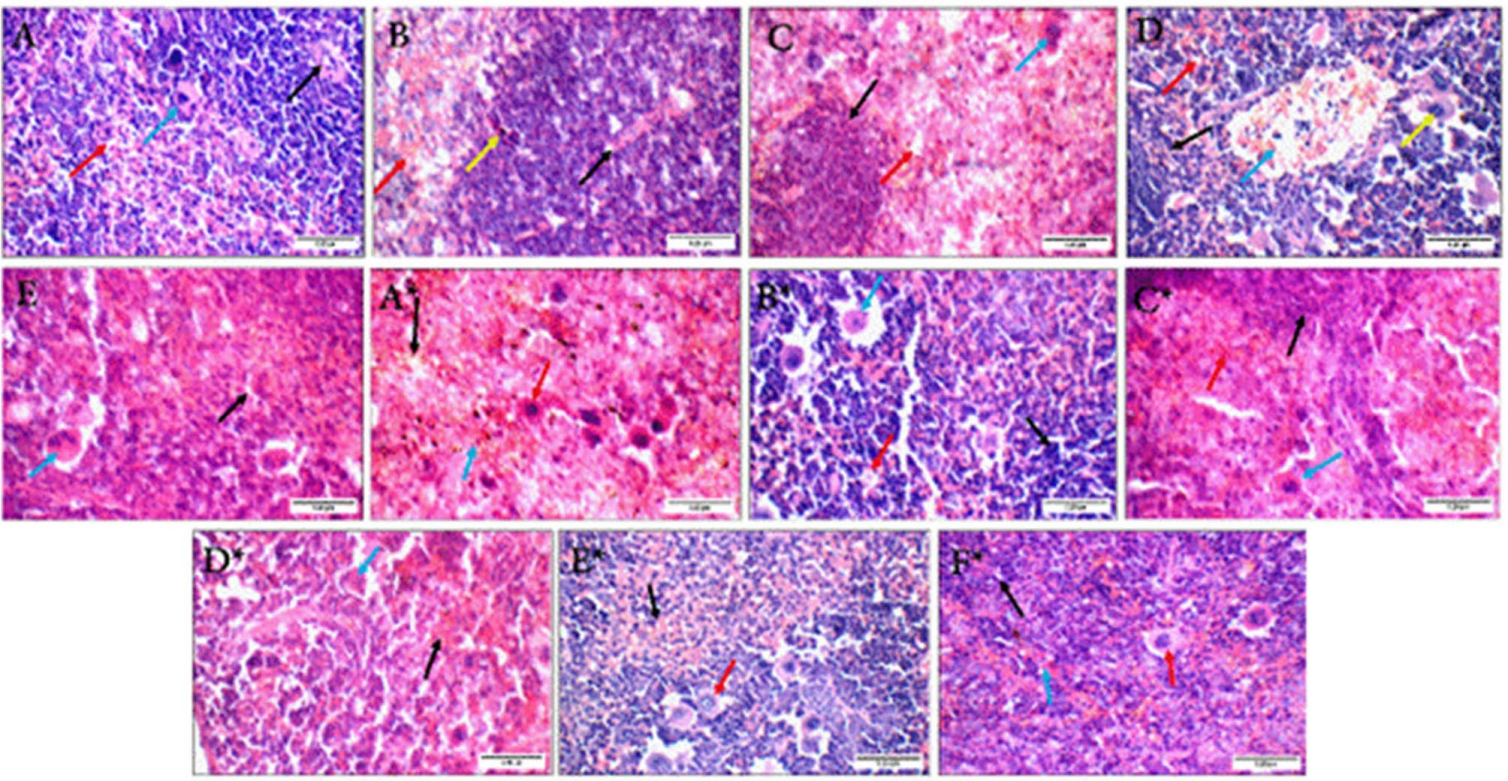
Spleen sections of non-infected groups: (**A**) control group showing average lymphoid follicles (white bulb) with central arterioles (black arrow), average blood sinusoids (red arrow), and scattered giant cells (blue arrow), (**B**) BV group showing average lymphoid follicles (black arrow) with scattered apoptotic cells (yellow arrow), and mildly congested blood sinusoids (red arrow), (**C**) irradiated BV group showing average lymphoid follicles (black arrow), average blood sinusoids (red arrow), and excess giant cells (blue arrow), (**D**) CS-NPs group showing average lymphoid follicles (black arrow), average blood sinusoids (red arrow), mildly congested blood vessels (blue arrow), and excess giant cells (yellow arrow), (**E**) BV-loaded CS-NPs group showing average blood sinusoids (black arrow), and scattered giant cells (blue arrow). Spleen sections of infected groups with *S. aureus*: (A*) no treatment group showing markedly congested blood sinusoids (red arrow), scattered apoptotic cells (blue arrow), and excess giant cells (black arrow), (B*) BV group showing average lymphoid follicles (black arrow), average blood sinusoids (red arrow), and excess giant cells (blue arrow), (C*) irradiated BV group showing small-sized lymphoid follicles (black arrow), congested blood sinusoids (red arrow), and scattered giant cells (blue arrow), (D*) CS-NPs showing mildly congested blood sinusoids (black arrow), and scattered giant cells (blue arrow), (E*) BV-loaded CS-NPs showing average blood sinusoids (black arrow), and excess giant cells (red arrow), (F*) antibiotic group showing average lymphoid follicles (black arrow), average blood sinusoids (blue arrow), and excess giant cells (red arrow) (H&E × 400). BV: Bee venom, CS-NPs: Chitosan nanoparticles, BV-loaded CS-NPs: Bee venom loaded Chitosan nanoparticles, *S. aureus: Staphylococcus aureus,* H&E: Hematoxylin and Eosin.

For the infected groups, the spleen sections of the no-treatment group showed markedly congested blood sinusoids, scattered apoptotic cells, and excess giant cells. The congestion decreased in the gamma-irradiated BV and CS-NPs groups, while it completely disappeared in the BV, BV-loaded CS-NPs, and antibiotic-treated groups. All groups showed average-sized lymphoid follicles except for the gamma-irradiated BV, and the presence of excess giant cells was also observed in all groups except for the gamma-irradiated BV and CS-NPs groups (Fig. 11).

#### Histopathological analysis of thymus samples

All of the non-infected groups showed thymus sections similar to the control but with some minor variations, as the BV group showed mild congestion in blood vessels, while both the CS-NPs and BV-loaded CS-NPs showed scattered apoptotic thymocytes in the cortex.

**Figure 12:**
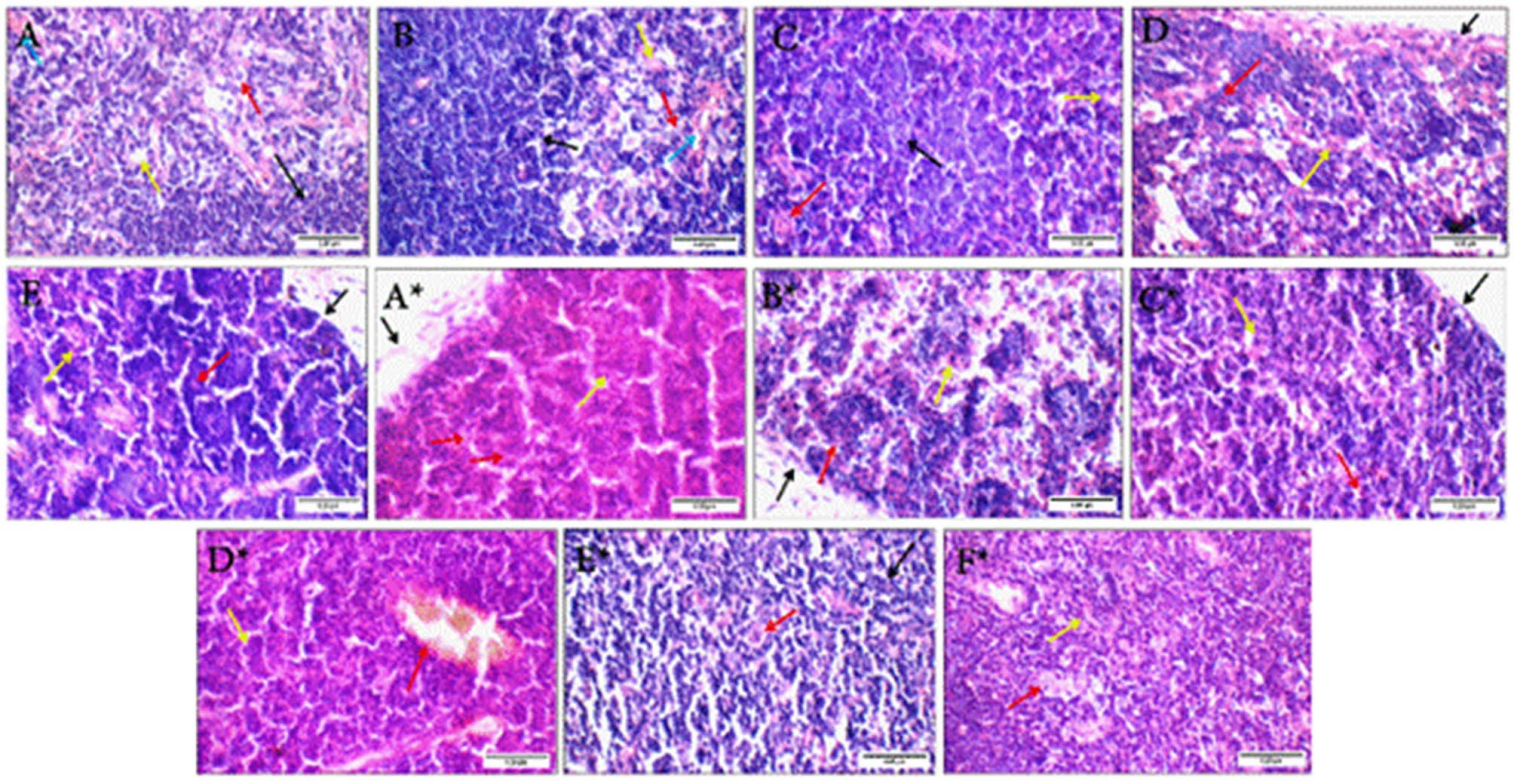
Thymus sections of non-infected groups: (**A**) control group showing average cortex with average thymocytes (black arrow), and average medulla with average reticular epithelial cells (red arrow), average Hassall's corpuscles (yellow arrow), and average blood vessels (blue arrow), (**B**) BV group showing cortex with average thymocytes (black arrow), and medulla with average reticular epithelial cells (red arrow), average Hassall's corpuscles (yellow arrow), and mildly congested blood vessels (blue arrow), (**C**) irradiated BV group showing cortex with average thymocytes (black arrow), and medulla with average reticular epithelial cells (red arrow) and average Hassall's corpuscles (yellow arrow), (**D**) CS-NPs group showing average capsule (black arrow), thin cortex with scattered apoptotic thymocytes (red arrow), and medulla with excess reticular epithelial cells (yellow arrow), (**E**) BV-loaded CS-NPs group showing average capsule (black arrow), cortex with scattered apoptotic thymocytes (red arrow), and medulla with average reticular epithelial cells (yellow arrow). Thymus sections of infected groups with *S. aureus*: (A*) no treatment group showing detached capsule (black arrow), cortex with markedly apoptotic thymocytes (red arrow), and average reticular epithelial cells (yellow arrow), (B*) BV group showing average capsule (black arrow), thin cortex with scattered apoptotic thymocytes (red arrow), and medulla with excess reticular epithelial cells (yellow arrow), (C*) irradiated BV group showing average capsule (black arrow), cortex with scattered apoptotic thymocytes (red arrow), and medulla with excess reticular epithelial cells (yellow arrow), (D*) CS-NPs group showing cortex with average thymocytes (yellow arrow), and medulla with mildly congested blood vessels (red arrow), (E*) BV-loaded CS-NPs group showing cortex with average thymocytes (black arrow), and medulla with average reticular epithelial cells (red arrow), (F*) antibiotic group showing cortex with scattered apoptotic thymocytes (yellow arrow), and medulla with average reticular epithelial cells (red arrow) (H&E × 400). BV: Bee venom, CS-NPs: Chitosan nanoparticles, BV-loaded CS-NPs: Bee venom loaded Chitosan nanoparticles, *S. aureus: Staphylococcus aureus,* H&E: Hematoxylin and Eosin.

Meanwhile, there were great variations in the thymus sections of infected groups. The untreated group showed a detached capsule, cortex with markedly apoptotic thymocytes, and average reticular epithelial cells. Excess reticular cells in the medulla were observed in all other groups except BV-loaded CS-NPs. BV, gamma-irradiated BV, and antibiotic-treated groups also showed cortex with scattered apoptotic thymocytes. The BV-loaded CS-NPs treated group sections were very similar to the control with average cortex, average thymocytes, and average medulla with average reticular cells (Fig. 12).

## Discussion

The number of ARB strains increases annually, rendering most antibiotics ineffective, leading to it being one of the largest causes of deathaused by continuous antibiotic abuse^4^. Honeybee venom extracted from *A. mellifera* can potentially be a therapeutic agent for different diseases. Nevertheless, it is a toxin that can lead to several consequences^24^. So, our goal is to study its therapeutic effect by testing the antibacterial activity while eliminating some of its cytotoxicity by treating BV with gamma radiation and loading the BV on CS-NPs.

The CS-NPs were prepared by the ionic gelation method, which depends mainly on electrostatic interactions. TEM has established the hypothesis of successfully incorporating BV on CS-NPs due to the size variation between the BV-loaded CS-NPs and unloaded CS-NPs. This was further confirmed by the results of the release rate and EE showing an excellent BV uptake by the CS-NPs. Furthermore, the surface charge of NPs decreased upon loading of BV, which was later confirmed by the FTIR results. All of these results were in accordance with previous studies^25,26^.

BV is composed of a complex of proteins that several methods could characterize. Exposing BV to gamma radiation leads to changes in the conformation of those components, which leads to the molecular weight change of BV. So, FTIR analysis was performed for further confirmation of different forms of BV. The comparative FTIR analysis of BV and irradiated BV proved the purity of obtained venoms, as there was no presence of any bands other than those of the venom itself. In addition, all of the peaks shown in the spectra of BV were relevant to its actual composition. Alteration of those bands in the spectra of gamma-irradiated BV proved the change in the functional groups caused by radiation. The impact of those changes is later analyzed by comparing the antibacterial activity and cytotoxic effect of both BV and irradiated BV^27^.

The comparative FTIR spectra of CS-NPs and BV-loaded CS-NPs also confirmed the uptake of BV by NPs, showing the specific bands for nanoparticles in both the unloaded BV-loaded CS-NPs spectra, in addition to the sharp band for BV at 1642 cm^−1^ indicating that BV molecules were absorbed on the NPs' surface. Phosphoric ions were also present. Consequently, the NPs FTIR spectra were compatible with the result of a phosphate-modified CS film. The phosphate groups of Na-TPP were attached to amino groups of CS molecules in NPs. TPP served as a cross-linker during preparation, forming additional hydrogen bonds with amino groups on CS and BV.

The in vitro disc diffusion method tested the difference between the antibacterial activity of irradiated BV and BV-loaded CS-NPs. It showed that the loaded NPs have more antibacterial activity than irradiated BV according to their respective diameters of the inhibition zones.

Hematological investigations showed no significant results in different mice groups compared to the control group; however, they show a relevant decrease in WBCs, which indicates the infection and liver damage in infected groups^28^, in non-infected groups, WBCs decreased due to the cytotoxicity of BV and CS as an immune response against CS-NPs^29^. Also, treated infected groups, especially those treated with CS-NPs, with BV-loaded CS-NPs or with antibiotics, showed results similar to the normal range, which means they have suitable antibacterial activity with low cytotoxicity.

In the non-infected groups, the liver functions showed a decrease in ALT levels of BV-treated mice, indicating the anti-inflammatory effect of BV^30^ and an increase in AST levels due to the presence of PLA2, which leads to liver injury^31^. These results were consistent with the liver sections, which showed mildly vacuolated cytoplasm as evidence of liver injury and are consistent with previous studies^32^. The increase in ALT and AST levels in the irradiated BV-treated group was due to the alteration in the protein content structure of BV due to radiation which affected its anti-inflammatory effect. The increased levels of ALT and AST in CS-NPs and BV-loaded CS-NPs treated groups indicate the protective role of the NPs on the hepatocytes^33^ and are also supported by average hepatocytes in liver sections. After the bacterial infection, ALT and AST levels increased in all infected treated groups compared to the control group. Histopathological sections of liver results also showed a mild inflammatory infiltrate in all infected treated groups, leading to fibrogenesis^34^.

The kidney is the second organ exposed to infection. Kidney sections of the control group showed average glomeruli with average Bowman's spaces, average proximal tubules with preserved brush borders, average distal tubules, and average interstitial blood vessels. These histopathological results started to change in the BV-injected group showing mildly edematous epithelial lining and markedly congested interstitial blood vessels, leading to increased renal interstitial pressure affecting the entire capillary bed and tubules possibly causing local hypoxia^35^. The inflammatory response decreased in irradiated BV and CS-NPs injected groups showing mildly congested interstitial blood vessels. Meanwhile, the BV-loaded CS-NPs injected group showed a huge change in its kidney sections with scattered apoptotic epithelial lining and markedly dilated congested blood vessels. These histopathological results of the kidney were supported by kidney function results showing a slight increase in creatinine levels and a decrease in urea levels.

In the case of infected groups with *S. aureus*, the treatment with irradiated BV showed atrophied glomeruli with widened spaces and scattered apoptotic epithelial lining. The irradiated BV-treated group showed a reduction in urea levels and a high increase in creatinine levels as an indicator of severe bacterial infection with a high cytotoxic effect of irradiated BV^36,37^. The cytotoxicity began to decrease in the BV-treated group, indicating a decrease in urea and creatinine levels, while increasing levels of creatinine and decreasing urea levels in the BV-loaded CS-NPs treated group. This slight increase in creatinine levels indicated less severity of BV-loaded CS-NPs than irradiated BV. Take the results in line with the results of the histopathological samples of kidney sections, which were clarified in the case of the treatment with BV or BV-loaded CS-NPs showing only mildly congested interstitial blood vessels.

Spleen and thymus are two of the most important immune-regulatory organs^38,39^; previous studies proved that BV is a double-edged sword and could either trigger or suppress inflammatory and immune responses, the optimum treatment of BV would have an antibacterial effect without causing an allergic reaction^40^. Analysis of histological sections for the groups relative to their corresponding IL-6 levels showed that the control group had average spleen and thymus tissues. IL-6 levels of the non-infected groups were all slightly similar to the control with the injection and the BV-loaded CS-NPs groups being the closest to the control and the irradiated BV-treated group the most deviated, and this was further shown in the histology samples with the presence of excess giant cells in the spleen sections of irradiated BV injected group and a less amount of them in the BV-loaded CS-NPs. These giant cells result from numerous macrophage fusions due to high inflammation^41^. It was also notable that the CS-NPs might have induced an inflammatory response as it resulted in mild congestion and the presence of excess giant cells in the spleen and cortical apoptosis in the thymus, which is usually a result of infections or cytotoxicity^42^, we suggest that this could be due to the presence of impurities as CS-NPs normally play anti-inflammatory roles^43^.

Moreover, interleukin-6 (IL-6) which is a multifunctional cytokine that plays a crucial role in host defense due to its vast array of immunological and hematopoietic activities and its robust capacity to activate the acute phase response. The increase of IL-6 levels in addition to the thymic atrophy and damage of the spleen caused by *S. aureus* infection^42,44^, only seemed to decrease in the case of the BV-loaded CS-NPs treated group, which had average spleen and thymus sections and an IL-6 level that was very close to the negative control. All of the other drugs caused a significant increase in IL-6 levels in the infected groups. The irradiated BV also showed the most significant increase treated group, whose spleen sections along with the CS-NPs treated group sections, had mild congestions and excess giant cells. Thymus sections of the irradiated BV group also showed cortical apoptosis and increased medullary reticular cells. The overall summary of results suggested that, unlike radiation, loading the BV on CS-NPs gave it the most efficient antibacterial activity without triggering an inflammatory response or causing major damage to the thymus or spleen.

In conclusion, BV-loaded CS-NPs have shown relatively better results than the other alternatives, which are native BV and irradiated BV. The *In-vitro* study showed that the antibacterial effect of BV-loaded CS-NPs was greater than the alternatives. Furthermore, its cytotoxic effect was far less than the native and irradiated BV. These outcomes reinforce that BV-loaded CS-NPs may indeed be an adequate alternative to antibiotics and could replace them with some dedicated search.

## Data Availability

The datasets generated and/or analysed during the current study are not publicly available due but are available from the corresponding author on reasonable request.
